# 40-Hz transauricular vagal nerve stimulation rescues cognition of 9-month-old APP/PS1 mice via inhibiting hippocampal P2X7 receptor signaling

**DOI:** 10.3389/fnagi.2026.1766813

**Published:** 2026-03-13

**Authors:** Yutian Yu, Yu Wang, Shengnan Guo, Jinling Zhang, Ying Bai, Chang Liu, Xubin Liu, Xin Li, Xuejiao Jiang, Pengfei Liu, Jing Ling, Jiaying Zhao, Chunzhi Tang, Peijing Rong

**Affiliations:** 1 Beijing Shijitan Hospital, Capital Medical University, Beijing, China; 2 Ninth School of Clinical Medicine, Peking University, Beijing, China; 3 Institute of Acupuncture and Moxibustion, China Academy of Chinese Medical Sciences, Beijing, China; 4 School of Traditional Chinese Medicine, Capital Medical University, Beijing, China; 5 Medical College of Acu-Moxi and Rehabilitation, Guangzhou University of Chinese Medicine, Guangzhou, China; 6 Acupuncture Department, Chinese People’s Liberation Army General Hospital, Beijing, China; 7 College of Traditional Chinese Medicine, Inner Mongolia Medical University, Hohhot, China; 8 Department of Gynecology, Shenzhen Traditional Chinese Medicine Hospital, Shenzhen, China; 9 The Fourth Clinical Medical College of Guangzhou University of Chinese Medicine, Shenzhen, China; 10 Institute of Basic Research in Clinical Medicine, China Academy of Chinese Medical Sciences, Beijing, China

**Keywords:** 40-Hz, Alzheimer’s disease, cognition, frequency-specific, P2X7R signaling, transauricular vagal nerve stimulation

## Abstract

The lack of any viable therapy for Alzheimer’s disease (AD) evoked the study and utilization of neuromodulation. 40-Hz transauricular vagal nerve stimulation (taVNS) preliminarily showed effectiveness in mice with partial cognitive impairment in our previous work, yet 3 major problems remained unresolved. (1) Can 40-Hz taVNS rescue the cognition of mice exhibiting total cognitive impairment? (2) Are the cognition-rescuing effects of taVNS specific to 40 Hz stimulation? (3) Via P2X7R signaling? Thus, 3 parts were divided to address the above issues in this work using 9-month-old wild-type (WT) and APPswe/PS1dE9 (APP/PS1) mice. Behavioral examinations for cognition; western blotting (WB), enzyme-linked immunosorbent assays (ELISA) for Aβ load; WB and ELISA for the P2X7R signaling pathway; Nissl staining for neuroprotective effects were employed. 3 findings can be derived. (1) 40-Hz taVNS rescues cognition of the APP/PS1 mice aged 9 months (but is not effective in WT mice of the same age). (2) The cognition-rescuing effects of taVNS in APP/PS1 mice are frequency-specific, which is 40 Hz in this work (neither 8-Hz taVNS nor 80-Hz taVNS works). (3) The hippocampal P2X7R signaling is a critical mediator of the observed effects (inhibiting P2X7R had similar effects to 40-Hz taVNS, which counteracted activating P2X7R). Therefore, 40-Hz taVNS shows potential as a viable therapy option for AD, with the P2X7R being a prominent target.

## Introduction

Alzheimer’s disease (AD) is a progressive neurological ailment that deteriorates with age, impairing memory, spatial cognition, and reasoning. Epidemiological studies indicate that most AD patients are aged 65 or older (Jia et al., 2014; Huang et al., 2019). AD risk increases markedly with age, and society faces a growing duty to address its ramifications unless effective therapies are implemented.

There remains a dearth of efficacious treatments for AD. Most of the drugs approved by the U. S. FDA offer merely transient alleviation of symptoms associated with AD. Consequently, non-pharmacological therapies for AD are presently garnering interest, particularly with the application of neuromodulation methods.

Vagal nerve stimulation (VNS) is a novel neuromodulation approach with several neurological applications (Wang Y. et al., 2021). Clinical pilot investigations suggest that invasive VNS (iVNS) may improve cognitive performance in AD patients (Sjogren et al., 2002; Merrill et al., 2006). Anatomically, vagal afferent fibers reach the external auditory canals, enabling transauricular VNS (taVNS)—a non-invasive, affordable alternative that activates similarly to iVNS (HOU et al., 2022). Our prior clinical trial demonstrated that taVNS enhances cognition in mild cognitive impairment (MCI) patients (Wang et al., 2022), and animal work confirmed its benefits in early-stage AD mice (6-month-old APP/PS1 mice) (Yu et al., 2023). However, whether taVNS retains efficacy in advanced AD with complete cognitive impairment remains unknown—a critical gap given the progressive nature of the disease.

Notably, the selection of stimulation parameters (e.g., frequency) is pivotal for taVNS efficacy. While 20–30 Hz is commonly used in VNS (Thompson et al., 2021), gamma-frequency (40-Hz) sensory stimulation (GENUS) has shown remarkable Aβ clearance and cognitive rescue in AD models (Iaccarino et al., 2016; Adaikkan et al., 2019; Martorell et al., 2019; Murdock et al., 2024). Inspired by these findings, we previously applied continuous 40-Hz taVNS to early AD mice (Yu et al., 2023), yet two key questions persist: (1) Is 40-Hz truly superior to other frequencies? (2) Can its effects extend to advanced AD stages?

Although the etiology of AD is uncertain, it is linked to Aβ deposition, neuroinflammation, neurofibrillary tangles, and disease progression (Kaczmarczyk et al., 2018). Neuroinflammation and amyloid pathology spread tau, leading to brain malfunction and cognitive decline (Pascoal et al., 2021). Therefore, interventions that reduce both amyloid burden and neuroinflammation may be more beneficial than targeting each alone. 40-Hz taVNS achieves these effects (Yu et al., 2023).

The purinergic receptor family can be classified into 2 primary categories, namely P2X and P2Y. The P2X7 receptor, a constituent of the P2X family, is an ion channel receptor activated by ATP and is found in many regions of the CNS (Ni et al., 2020); it plays a vital role in neuroinflammation (Chen et al., 2021) and numerous neurological processes (Coddou et al., 2011). Multiple AD developmental routes depend on the P2X7R.

The P2X7R plays a necessary role in activating microglia by Aβ (Sanz et al., 2009). The P2X7R possesses the capability to regulate the synthesis of Aβ, and the investigation revealed that administering P2X7R antagonists resulted in a decrease of plaques in J20 mice (Diaz-Hernandez et al., 2012). The deficiency of the P2X7R, achieved using knock-out (KO) techniques, resulted in a cognitive improvement and a decrease in Aβ accumulation in one of the AD models (Martin et al., 2019). In our prior study, it was seen that the application of 40-Hz taVNS led to a simultaneous reduction in expressions of P2X7R and Aβ, which resulted in the initiation of a neuroprotective cascade reaction (Yu et al., 2023).

P2X7R promotes inflammation by activating the NLRP3 inflammasome (Adinolfi et al., 2018), which can also be triggered by Aβ (Halle et al., 2008). This activation initiates a downstream cascade leading to Caspase-1 production via cleavage of pro-Caspase-1 (Chen et al., 2021). The resulting generation of IL-1β and IL-18 (Zhu et al., 1999) creates a cerebral inflammatory environment that sustains microglial activation and chronic inflammation (Milner et al., 2021). Continuous NLRP3 activation in microglia impairs Aβ clearance, establishing a detrimental feedback loop between Aβ accumulation and microglial activation. Notably, genetic deletion of NLRP3 or Caspase-1 in an AD model with Aβ deposition reduced neuroinflammation, amyloid plaque load, and AD-related pathology, effectively preventing cognitive decline (Heneka et al., 2013; Tan et al., 2013; Heneka et al., 2014; Heneka et al., 2015a; Heneka et al., 2015b). Caspase-1 plays a key role in promoting inflammatory responses and cell death (Milner et al., 2021). The inflammasome-Caspase-1 complex cleaves cellular substrates (Guo et al., 2015; Boucher et al., 2018), processing pro-IL-1β and pro-IL-18 into their active forms to drive inflammation (Agostini et al., 2004; Martinon and Tschopp, 2004; Kanneganti et al., 2006; Mariathasan et al., 2006; Halle et al., 2008; Bryant and Fitzgerald, 2009; Martinon et al., 2009; Horvath et al., 2011; Wen et al., 2013; Lamkanfi and Dixit, 2014). Inhibiting Caspase-1 has also been shown to mitigate cognitive deficits and pathology in AD mice (Flores et al., 2018).

P2X7R activation can also trigger the activation of Nuclear factor-κB (NF-κB), which in turn stimulates the development and dissolution of inflammatory factors such as IL-1β and IL-18 (Korcok et al., 2004; Thawkar and Kaur, 2019; Wang et al., 2020). The creation and activation of the NLRP3 inflammasome require NF-κB as a transcriptional activator. Interconnections between NF-κB and NLRP3 pathways cause inflammatory mediator release and pyroptosis activation (Swanson et al., 2019). Moreover, pro-IL-1β and pro-IL-18, the cytoplasmic progenitors of IL-1β and IL-18, are produced by the pathway regulated by NF-κB (Hanslik and Ulland, 2020).

Inhibiting P2X7R suppresses neuroinflammation, making it an emerging AD treatment target (Huang et al., 2023). Our prior animal research showed that taVNS at a frequency of 40 Hz inhibits hippocampal P2X7R signaling in APP/PS1 mice aged 6 months (Yu et al., 2023), but no agonistic or antagonistic therapies were used to validate. Additionally, we are unaware of any investigations using P2X7R agonists and antagonists in APP/PS1 mice. The aforesaid reasons make this study groundbreaking.

**Figure 1:**
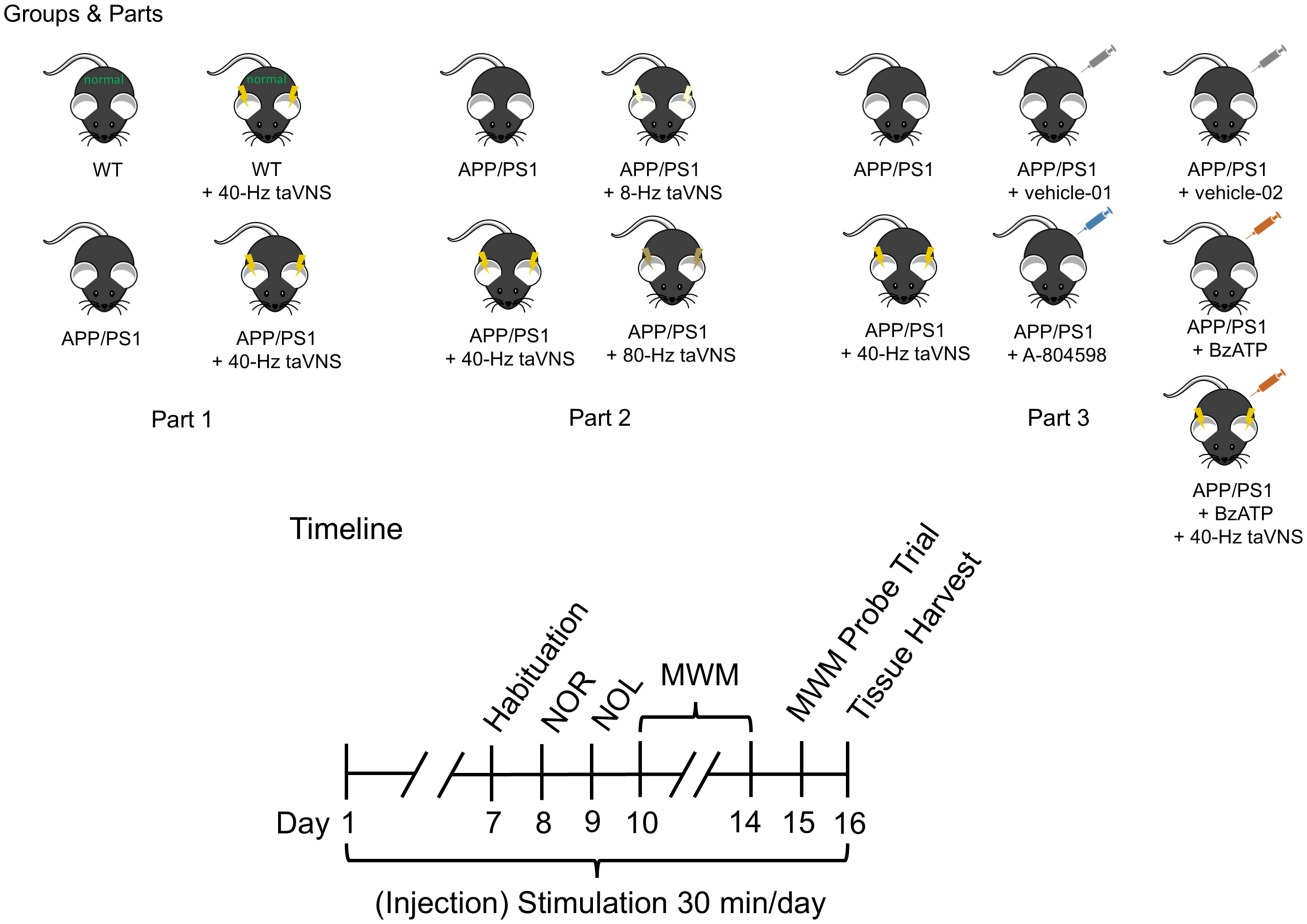
Illustration of experimental groups, parts, and timeline. There are 11 groups in total: (1) WT, (2) WT + 40 Hz-taVNS, (3) APP/PS1, (4) APP/PS1 + 40-Hz taVNS, (5) APP/PS1 + 8-Hz taVNS, (6) APP/PS1 + 80-Hz taVNS, (7) APP/PS1 + vehicle-01, (8) APP/PS1 + A-804598, (9) APP/PS1 + vehicle-02, (10) APP/PS1 + BzATP, and (11) APP/PS1 + BzATP + 40-Hz taVNS groups. The work includes 3 parts. Part 1 includes (1) WT, (2) WT + 40 Hz-taVNS, (3) APP/PS1, (4) APP/PS1 + 40-Hz taVNS groups; Part 2 includes (3) APP/PS1, (4) APP/PS1 + 40-Hz taVNS, (5) APP/PS1 + 8-Hz taVNS, (6) APP/PS1 + 80-Hz taVNS groups; Part 3 includes (3) APP/PS1, (4) APP/PS1 + 40-Hz taVNS, (7) APP/PS1 + vehicle-01, (8) APP/PS1 + A-804598, (9) APP/PS1 + vehicle-02, (10) APP/PS1 + BzATP, and (11) APP/PS1 + BzATP + 40-Hz taVNS groups. Timeline: Time points for daily (injection) stimulation; habituation phase of novel object recognition (NOR) and novel object location (NOL); training and testing of NOR; training and testing of NOL; acquisition training and probe trial of Morris water maze (MWM); and tissue harvest. Daily (injection) stimulation (taVNS at 8 Hz, 40 Hz, or 80 Hz) began on day 1 when the mice reached 9 months old and continued for a duration of 16 days. The (injection) stimulation was administered in the morning (8:00–12:00 a.m.), and behavioral tests were conducted in the afternoon (2:00–5:00 p.m.). On day 16, tissue was harvested in the afternoon (2:00–5:00 p.m.).

To address the aforementioned gaps, we pursued three major objectives in this work (Figure 1): (1) Validate 40-Hz taVNS efficacy in 9-month-old APP/PS1 mice with complete cognitive impairment; (2) Compare 8-Hz, 40-Hz, and 80-Hz taVNS to define frequency specificity; (3) Elucidate the role of P2X7R signaling via agonist/antagonist interventions. So, this study was divided into three parts. Part 1 replicates our prior research (Yu et al., 2023) and provides additional evidence supporting the cognition-ameliorating effects of 40-Hz taVNS on an advanced AD model. Part 2 partially mimicked the work by Tsai and colleagues (Martorell et al., 2019) and confirmed that the ameliorative effects of taVNS on AD mice were also frequency-specific (40 Hz). Part 3 is seminal; not only did we confirm that 40-Hz taVNS acts on APP/PS1 mice at 9 months of age via inhibiting hippocampal P2X7R signaling, but we also firstly confirmed the cognitive-improving effects of the P2X7R antagonist (A-804598) on 9-month-old APP/PS1 mice.

## Materials and methods

### Animals

18 wild-type (WT, C57BL/6) mice and 81 APPswe/PS1dE9 (APP/PS1) mice, all male, were procured from Gene & Peace Biotech Co., Ltd.^1^ at an average age of 8 months. The mice were placed in groups of 3 per enclosure, subjected to a regulated light/dark cycle of 12 h each, and kept at a consistent ambient temperature of 22 ± 1 °C. Water and food were freely available. The mice were first grouped by genotype and treatment into 11 groups: (1) WT, (2) WT + 40 Hz-taVNS, (3) APP/PS1, (4) APP/PS1 + 40-Hz taVNS, (5) APP/PS1 + 8-Hz taVNS, (6) APP/PS1 + 80-Hz taVNS, (7) APP/PS1 + vehicle-01, (8) APP/PS1 + A-804598, (9) APP/PS1 + vehicle-02, (10) APP/PS1 + BzATP, and (11) APP/PS1 + BzATP + 40-Hz taVNS. Each group consisted of 9 mice. Mice acclimatized for four weeks. As soon as the mice turned 9 months old, the experiment got underway. See Figure 1 for the experimental groups, parts, and timeline.

### Treating procedures

Sevoflurane at a 3–4% concentration was utilized to anesthetize the mice in this work. Untreated WT and APP/PS1 mice underwent 30 min of anesthesia without further treatment daily. Mice in the WT + 40-Hz taVNS, APP/PS1 + 40-Hz taVNS, APP/PS1 + 8-Hz taVNS, or APP/PS1 + 80-Hz taVNS groups were subjected to their respective taVNS under anesthesia. Mice in the APP/PS1 + vehicle-01 and the APP/PS1 + vehicle-02 group were administered injections with saline either intraperitoneally (vehicle-01) or intraventricularly (vehicle-02) and received 30 min of anesthesia daily. Mice in the APP/PS1 + A-804598 and the APP/PS1 + BzATP groups were administered injections of either A-804598 intraperitoneally or BzATP intraventricularly and received 30 min of anesthesia daily. In the APP/PS1 + BzATP + 40-Hz taVNS group, the initiation of 30 min of 40-Hz taVNS occurred one hour following the daily intraventricular delivery of BzATP.

### Timing

To minimize biological rhythms, the experimental regimen of medication injection and taVNS was conducted between 8:00 a.m. and 12:00 p.m. in the morning, from the first to the sixteenth day.

### Drug administration

A-804598 (a P2X7 receptor antagonist, 30 mg/kg/day, Ambeed, A270115) (Andrejew et al., 2020) and BzATP (a P2X7 receptor agonist, 30 nmol in 1 μL saline, Ambeed, A433956) (Müller and Namasivayam, 2022) were used in this investigation. An intraperitoneal injection of A-804598 or an intraventricular injection of BzATP was given daily to respective groups (Ribeiro et al., 2019; Deng et al., 2021).

### Transauricular vagal nerve stimulation (taVNS)

Initially, each mouse was anesthetized with 3–4% sevoflurane. According to earlier studies (Guo et al., 2020; Wang J.-Y. et al., 2021), a pair of magnetic electrodes with opposite polarity (+ and -) was placed adjacent to a custom-made metal ear splint that was 2 cm long, 0.3 cm wide, and 0.05 cm thick. The components were combined and affixed to the bilateral auricular concha. This structure allowed electrical current to cross the dermal layer and activate the auricular vagal nerve fibers (He et al., 2013; Wang J.-Y. et al., 2021). 30 min were spent on transauricular vagal nerve stimulation (taVNS). The intensity was 1.8 milliamperes, the pulse width was 0.5 milliseconds, and the frequency was 8, 40, or 80 Hz, depending on the group. Daily electroacupuncture was performed using Hwato SDZ-III equipment. A continuous waveform was used (Guo et al., 2020).

### Behavioral experiments

On days seven through fifteen, the behavioral examinations of 99 mice took place during the afternoon hours (2:00–5:00 p.m.), as depicted in Figure 1. And experimenters were blinded during behavioral testing.

### Novel object recognition (NOR)

As indicated (Leger et al., 2013), the NOR task required habituation before training and assessment the next day. The mice habituated in a 40-by-40-by-35-cm open testing arena on the seventh afternoon of the experiment. The habituation period lasted 5 min, during which the TSE Systems recorded distance and velocity in millimeters per second. On day eight, training and testing occurred. The mice were placed in a shared enclosure containing two identical items positioned in diagonally opposite corners for training purposes. After 20 s of playing with the objects within a 10-min, the mice were removed from the experimental container. After one hour, object memory was assessed using the same method as the training phase, except one object was replaced with a new one. They were observing the snout-object interaction documented item exploration. This was quantified using the object recognition index (ORI). To compute the ORI, divide the duration of interaction with the novel object (*T_novel_*) by the combined duration of engagement with the unfamiliar and familiar items. *ORI = T_novel_/(T_novel_ + T_familiar_).*

### Novel object location (NOL)

The NOL task was similar to the new object recognition task, except two identical objects were utilized for training and assessment. During testing, one item was moved to a new location. NOL testing was on day nine.

### Morris water maze (MWM)

The MWM evaluates spatial learning and memory capabilities. We used a 120-centimeter-circle, 50-centimeter-deep tank. We divided the tank into four quadrants: 1, 2, 3, and 4. A 1-centimeter-deep platform was in quadrant 3, in the target region. The test includes acquisition training and probe trials. A 24-h delay separated the NOL test from the mice’s five-day training phase (days 10–14). The mice were allotted 60 s to locate the platform and 10 s to remain on it. We measured mean speed and escape latency. Mice unable to locate the platform within 60 s were thereafter positioned on it for 10 s. On day 15, the inquiry trial began. The mice were given 60 s to swim without the platform in quadrant 1, across from quadrant 3. The number of platform crossings and quadrant 3 times were recorded.

### Enzyme-linked immunosorbent assay (ELISA)

On day 16, six mice per group were anesthetized with 6% sevoflurane, and hippocampi were harvested. Hippocampal levels of A*β*1-40, Aβ1-42, NF-κB, pro-IL-1β, pro-IL-18, IL-1β, and IL-18 were quantified using commercial kits: Aβ1-40 (EIA-2463) and Aβ1-42 (EIA-2125) from Shanghai Elisa Biotech; NF-κB (JL20372), pro-IL-1β (JL51668), pro-IL-18 (JL53723), IL-1β (JL18442), and IL-18 (JL20253) from Shanghai JONLN Reagent. Assays were performed with a BioTek-ELx800 microplate reader following manufacturer protocols.

### Western blotting (WB)

Hippocampal Aβ42, P2X7R, NLRP3, and Caspase-1 concentrations were analyzed via WB (*n* = 6 per group). Tissue samples were homogenized in ice-cold RIPA buffer, centrifuged at 15,000 rpm for 20 min, and supernatants were obtained for protein quantification. The homogenate proteins were fractionated using SDS-PAGE and subsequently transferred to PVDF membranes. Following a 1-h blocking period with 5% skim milk, membranes were incubated overnight at 4 °C with primary antibodies: anti-β-amyloid (ab201060, Abcam), anti-P2X7R (sc-134224, Santa Cruz), anti-NLRP3 (ab214185, Abcam), and anti-Caspase-1 (ab1872, Abcam). Following TBST washes, membranes were probed with HRP-conjugated secondary antibodies (goat anti-mouse IgG ab6789 or goat anti-rabbit IgG ab6721, Abcam) for 1 h at ambient temperature. ECL (#34080, ThermoFisher SCIENTIFIC) was used to visualize protein bands, and the images were transferred on hyperfilm. The blots were subjected to a stripping process and then re-probed using a loading control antibody. The loading control antibody used was either an anti-beta Tubulin antibody (ab179513, Abcam) or an anti-glyceraldehyde-3 phosphate dehydrogenase antibody (ab181602 Abcam). The protein bands’ gray values were measured using the ImageJ program. Ultimately, these values were standardized to the gray values of the control bands.

### Histological evaluations

On the sixteenth afternoon, the 3 remaining mice in each group were anesthetized with 6% sevoflurane and flushed with 0.9% physiological saline. After fixing the mice in 4% paraformaldehyde, their brains were swiftly removed. For a long time, the specimens were in 4% paraformaldehyde at 4 °C. They were then placed in a 30% sucrose solution at the same temperature until they sank. Mouse brain coronal slices were taken using a freezing microtome at a thickness of 30 μm.

### Nissl staining

Three 5-min washes with 1% PBS were performed on brain slices from 33 mice (*n* = 3 per group). After that, 1% toluidine blue was used to color the brain slices for an hour at 55 °C. The slices were subsequently rinsed twice in 1% PBS for 5 min. Following attachment to glass slides, the brain slices were desiccated in a 37 °C incubator, subjected to a gradient of alcohol for dehydration, and subsequently cleansed with xylene. The parts were covered with neutral gum and kept in a place with good airflow and temperature control.

### Scanning

Pannoramic MIDI slide scanners from 3DHISTECH Ltd. in Budapest, Hungary, were used to visualize brain slices. These photos were then analyzed using Pannoramic Viewer Software.

### Statistical analysis

Statistical analysis was done with GraphPad Prism 10. The escape time from the MWM test was evaluated using a two-way ANOVA, revealing a statistically significant impact, which led to additional Bonferroni *post hoc* testing. The residual data were examined utilizing one-way ANOVA, succeeded by Tukey’s post hoc analyses. A significance threshold of 0.05 was used. Results are expressed as mean values accompanied by standard deviations.

## Results

### The signaling of P2X7R is crucial in the cognitive-rescuing effects to 9-month-old APP/PS1 mice by 40-Hz taVNS

#### 40-Hz taVNS rescues cognition of 9-month-old APP/PS1 mice

**Figure 2:**
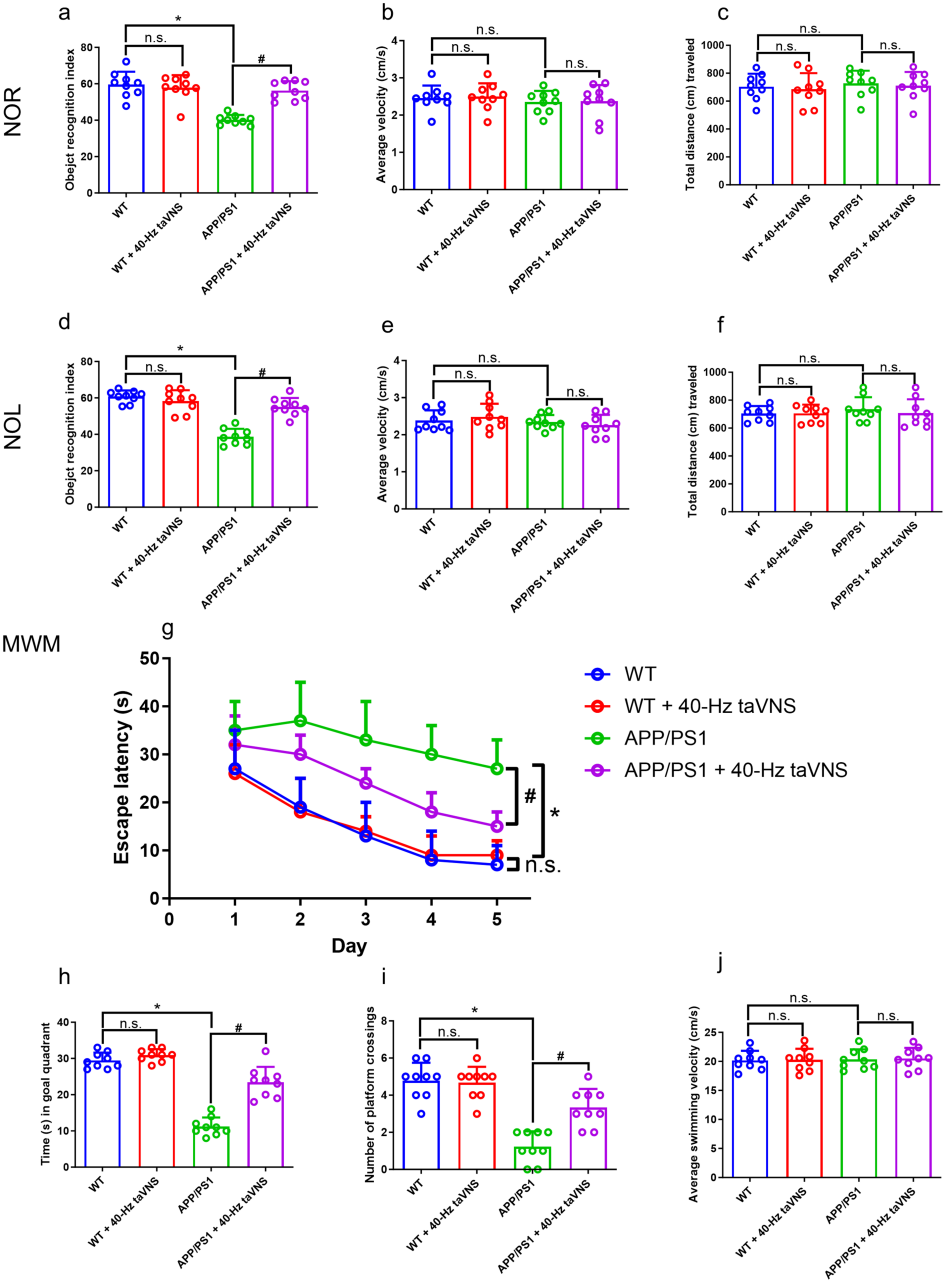
The results of the novel object recognition (NOR), novel object location (NOL), and Morris water maze (MWM) tests in part 1 of the study. **(a)** The recognition index of the NOR test (*n* = 9 per group, WT vs. WT + 40-Hz taVNS, n.s., not significant, *p* > 0.05; WT vs. APP/PS1, *p < 0.05; APP/PS1 vs. APP/PS1 + 40-Hz taVNS, #*p* < 0.05). **(b)** The average velocity (cm/s) during the NOR test (*n* = 9 per group, WT vs. WT + 40-Hz taVNS, WT vs. APP/PS1, and APP/PS1 vs. APP/PS1 + 40-Hz taVNS, n.s., not significant, *p* > 0.05). **(c)** The cumulative distance (cm) traveled throughout the NOR test (*n* = 9 per group, WT vs. WT + 40-Hz taVNS, WT vs. APP/PS1, and APP/PS1 vs. APP/PS1 + 40-Hz taVNS, n.s., not significant, *p* > 0.05). **(d)** The recognition index of the NOL test (*n* = 9 per group, WT vs. WT + 40-Hz taVNS, n.s., not significant, *p* > 0.05; WT vs. APP/PS1, *p < 0.05; APP/PS1 vs. APP/PS1 + 40-Hz taVNS, #*p* < 0.05). **(e)** The average velocity (cm/s) during the NOL test (*n* = 9 per group, WT vs. WT + 40-Hz taVNS, WT vs. APP/PS1, and APP/PS1 vs. APP/PS1 + 40-Hz taVNS, n.s., not significant, *p* > 0.05). **(f)** The cumulative distance (cm) traveled throughout the NOL test (*n* = 9 per group, WT vs. WT + 40-Hz taVNS, WT vs. APP/PS1, and APP/PS1 vs. APP/PS1 + 40-Hz taVNS, n.s., not significant, *p* > 0.05). **(g)** Escape latencies in the MWM (*n* = 9 per group, WT vs. WT + 40-Hz taVNS, n.s., not significant, *p* > 0.05; WT vs. APP/PS1, *p < 0.05; APP/PS1 vs. APP/PS1 + 40-Hz taVNS, #*p* < 0.05). **(h)** Time spent swimming in the goal quadrant during the probe trial (*n* = 9 per group, WT vs. WT + 40-Hz taVNS, n.s., not significant, *p* > 0.05; WT vs. APP/PS1, *p < 0.05; APP/PS1 vs. APP/PS1 + 40-Hz taVNS, #*p* < 0.05). **(i)** Number of platform crossings during the probe trial (*n* = 9 per group, WT vs. WT + 40-Hz taVNS, n.s., not significant, *p* > 0.05; WT vs. APP/PS1, *p < 0.05; APP/PS1 vs. APP/PS1 + 40-Hz taVNS, #*p* < 0.05). **(j)** Average swimming velocity (cm/s) during MWM (*n* = 9 per group, WT vs. WT + 40-Hz taVNS, WT vs. APP/PS1, and APP/PS1 vs. APP/PS1 + 40-Hz taVNS, n.s., not significant, *p* > 0.05).

Compared to WT controls, APP/PS1 mice displayed substantial impairment in both NOR and NOL tasks (Figures 2a,d). However, 40-Hz taVNS effectively reversed these cognitive deficits (Figures 2a,d). Importantly, the improved cognition was not attributable to changes in locomotor activity, as no differences in movement speed or distance traveled were observed between groups (Figures 2b,c,e,f).

In comparison to APP/PS1 mice, WT mice with or without 40-Hz taVNS showed considerably shorter escape latencies throughout training (Figure 2g). Continuous 40-Hz taVNS significantly shortened APP/PS1 mice’s escape time (Figure 2g). In the MWM probe trial, APP/PS1 mice subjected to taVNS at 40 Hz exhibited a considerably greater duration in the target quadrant and a higher frequency of platform location crossings compared to untreated controls (Figures 2h,i). No similar effects were detected in WT controls (Figures 2h,i). All four experimental groups had similar swimming speeds (Figure 2j). The results indicate that the cognitive changes seen in the APP/PS1 mice undergoing taVNS at a frequency of 40 Hz were not attributable to differences in locomotor activity.

#### 40-Hz taVNS reduces the accumulation of amyloid in 9-month-old APP/PS1 mice

**Figure 3:**
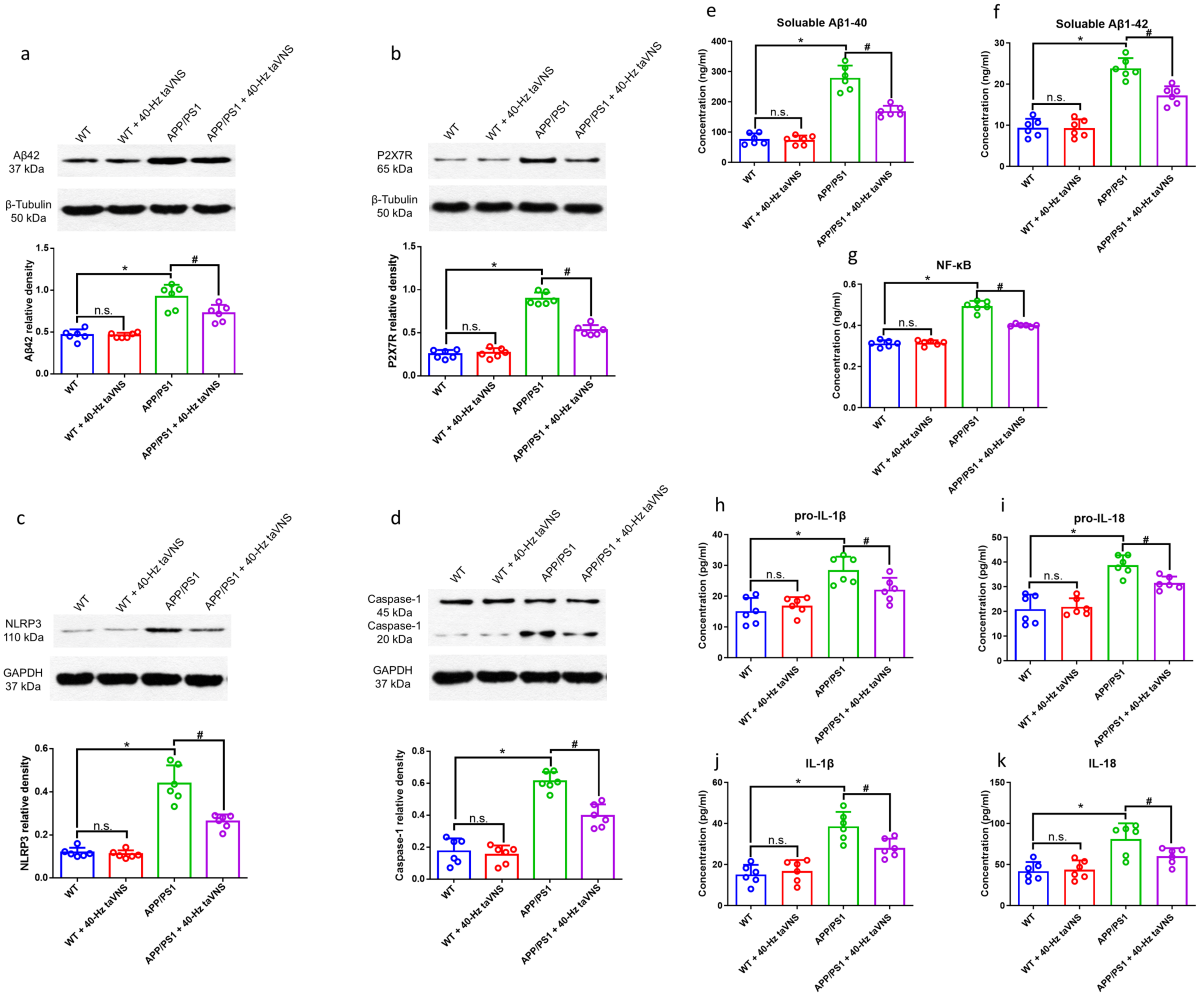
Hippocampal Aβ42, P2X7R, NLRP3, Caspase-1 expression, and Aβ1-40, Aβ1-42, NF-κB, pro-IL-1β, pro-IL-18, IL-1β, IL-18 levels in Part 1 of the study. WB results show hippocampal Aβ42 **(a)**, P2X7R **(b)**, NLRP3 **(c)**, and Caspase-1 (20 kDa) **(d)** expression. *n* = 6 per group, WT vs. WT + 40 Hz taVNS, n.s., not significant, *p* > 0.05; WT vs. APP/PS1, *p < 0.05; APP/PS1 vs. APP/PS1 + 40-Hz taVNS, #*p* < 0.05. ELISA results show soluble Aβ1-40 **(e)** and soluble Aβ1-42 **(f)**, NF-κB **(g)**, pro-IL-1β **(h)**, pro-IL-18 **(i)**, IL-1β **(j)** and IL-18 **(k)** levels in the hippocampi of the mice. *n* = 6 per group, WT vs. WT + 40-Hz taVNS, n.s., not significant, *p* > 0.05; WT vs. APP/PS1, *p < 0.05; APP/PS1 vs. APP/PS1 + 40 Hz taVNS, #*p* < 0.05.

40-Hz taVNS not only lowered hippocampal Aβ42 levels (Figure 3a) but also decreased soluble Aβ1-40 and Aβ1-42 (Figures 3e,f) in APP/PS1 mice.

#### 40-Hz taVNS inhibits P2X7R signaling in 9-month-old APP/PS1 mice

WB results demonstrated that APP/PS1 mice exhibited elevated hippocampal concentrations of P2X7R, NLRP3, and Caspase-1 (20 kDa) compared to WT controls (Figures 3b–d). Remarkably, 40-Hz taVNS decreased these protein levels, indicating suppression of the P2X7R signaling pathway (Figures 3b–d). In WT mice, such effects were absent (Figures 3b–d).

To further validate the role of P2X7R signaling, we measured downstream inflammatory mediators. ELISA results confirmed that 40-Hz taVNS suppressed hippocampal levels of NF-κB, pro-IL-1β/IL-1β, and pro-IL-18/IL-18 in APP/PS1 mice, aligning with the observed reduction in P2X7R pathway activity (Figures 3g–k).

#### 40-Hz taVNS offers neuroprotective advantages

Neuron structural integrity was assessed using Nissl staining (Kreutzberg, 1984; Luo et al., 2020). Compared to the untreated mice, hippocampal neurons exposed to taVNS at 40 Hz had better-ordered Nissl bodies and darker appearances (Supplementary Figure 1). Thus, both WT and APP/PS1 mice showed neuroprotective advantages from taVNS at 40 Hz.

### The efficacy of taVNS in APP/PS1 mice aged 9 months is limited to 40 Hz

#### The cognition-rescuing effects of taVNS in APP/PS1 mice aged 9 months are limited to 40-Hz stimulation

**Figure 4:**
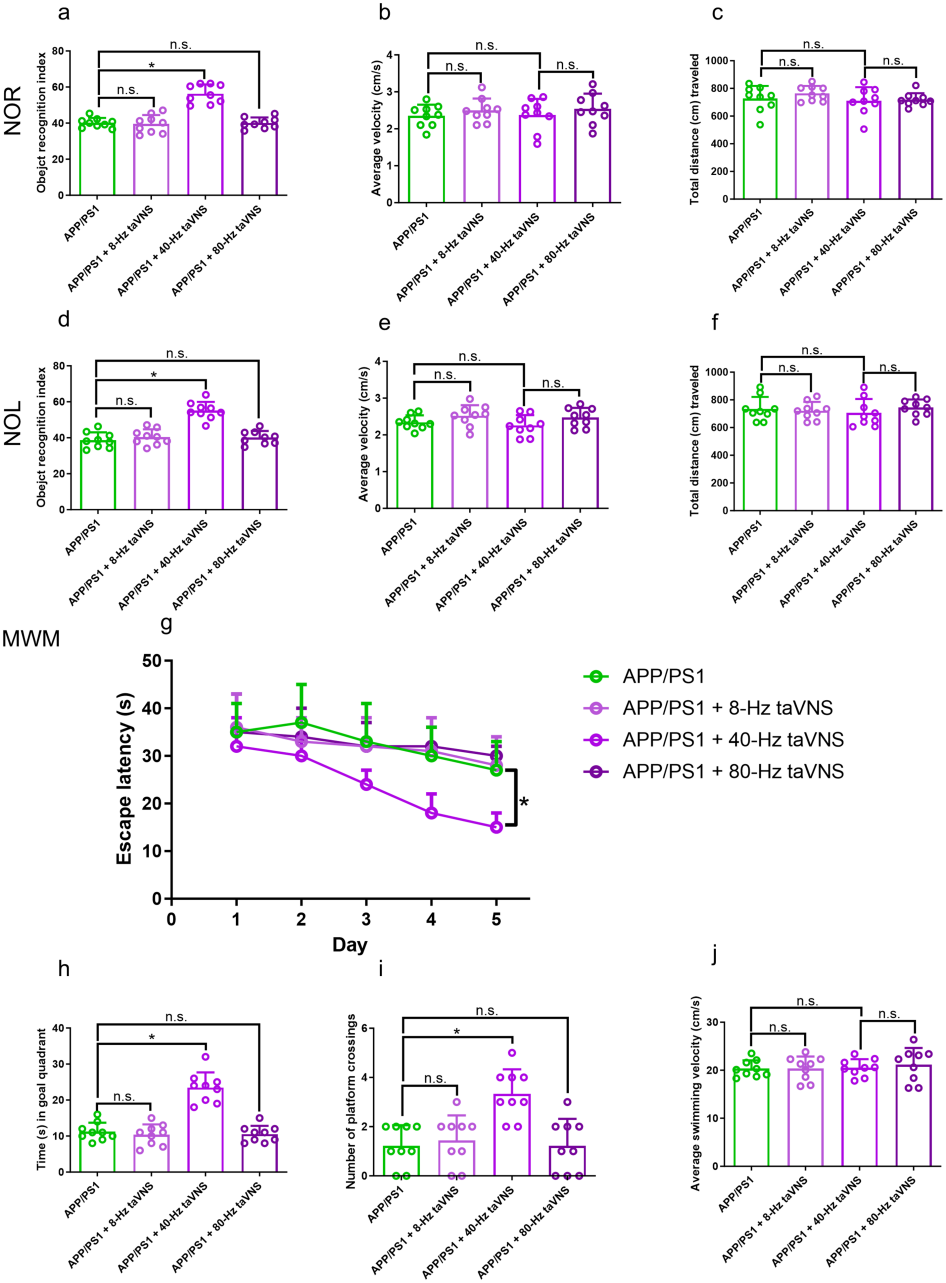
The results of the NOR, NOL, and MWM tests in Part 2 of the study. **(a)** The recognition index of the NOR test (*n* = 9 per group, APP/PS1 vs. APP/PS1 + 40-Hz taVNS, *p < 0.05; APP/PS1 vs. APP/PS1 + 8-Hz taVNS and APP/PS1 vs. APP/PS1 + 80-Hz taVNS, n.s., not significant, *p* > 0.05). **(b)** The average velocity (cm/s) during the NOR test (*n* = 9 per group, APP/PS1 vs. APP/PS1 + 8-Hz taVNS, APP/PS1 vs. APP/PS1 + 40-Hz taVNS, and APP/PS1 vs. APP/PS1 + 80-Hz taVNS, n.s., not significant, *p* > 0.05). **(c)** The cumulative distance (cm) traveled throughout the NOR test (*n* = 9 per group, APP/PS1 vs. APP/PS1 + 8-Hz taVNS, APP/PS1 vs. APP/PS1 + 40-Hz taVNS, and APP/PS1 vs. APP/PS1 + 80-Hz taVNS, n.s., not significant, *p* > 0.05). **(d)** The recognition index of the NOL test (*n* = 9 per group, APP/PS1 vs. APP/PS1 + 40-Hz taVNS, *p < 0.05; APP/PS1 vs. APP/PS1 + 8-Hz taVNS and APP/PS1 vs. APP/PS1 + 80-Hz taVNS, n.s., not significant, *p* > 0.05). **(e)** The average velocity (cm/s) during the NOL test (*n* = 9 per group, APP/PS1 vs. APP/PS1 + 8-Hz taVNS, APP/PS1 vs. APP/PS1 + 40-Hz taVNS, and APP/PS1 vs. APP/PS1 + 80-Hz taVNS, n.s., not significant, *p* > 0.05). **(f)** The cumulative distance (cm) traveled throughout the NOL test (*n* = 9 per group, APP/PS1 vs. APP/PS1 + 8-Hz taVNS, APP/PS1 vs. APP/PS1 + 40-Hz taVNS, and APP/PS1 vs. APP/PS1 + 80-Hz taVNS, n.s., not significant, *p* > 0.05). **(g)** Escape laten cies in the MWM (*n* = 9 per group, APP/PS1 vs. APP/PS1 + 40-Hz taVNS, *p < 0.05). **(h)** Time spent swimming in the goal quadrant during the probe trial (*n* = 9 per group, APP/PS1 vs. APP/PS1 + 8-Hz taVNS and APP/PS1 + 80-Hz taVNS, n.s., not significant, *p* > 0.05; APP/PS1 vs. APP/PS1 + 40 Hz taVNS, *p < 0.05). **(i)** Number of platform crossings during the probe trial (*n* = 9 per group, APP/PS1 vs. APP/PS1 + 8-Hz taVNS and APP/PS1 + 80-Hz taVNS, n.s., not significant, *p* > 0.05; APP/PS1 vs. APP/PS1 + 40-Hz taVNS, *p < 0.05). **(j)** Average swimming velocity (cm/s) during MWM (*n* = 9 per group, APP/PS1 vs. APP/PS1 + 8-Hz taVNS, APP/PS1 vs. APP/PS1 + 40-Hz taVNS, and APP/PS1 vs. APP/PS1 + 80-Hz taVNS, n.s., not significant, *p* > 0.05).

Among the evaluated frequencies, only 40-Hz taVNS significantly corrected cognitive deficiencies in APP/PS1 mice, which demonstrated better performance in NOR, NOL, and MWM tests (Figures 4a,d,g,h,i). In contrast, stimulation at 8 Hz or 80 Hz failed to elicit significant behavioral improvements (Figures 4a,d,g,h,i). Locomotor activity remained unaffected across all groups (Figures 4b,c,e,f,j).

#### 40-Hz taVNS specifically reduces the accumulation of amyloid in APP/PS1 mice aged 9 months

**Figure 5:**
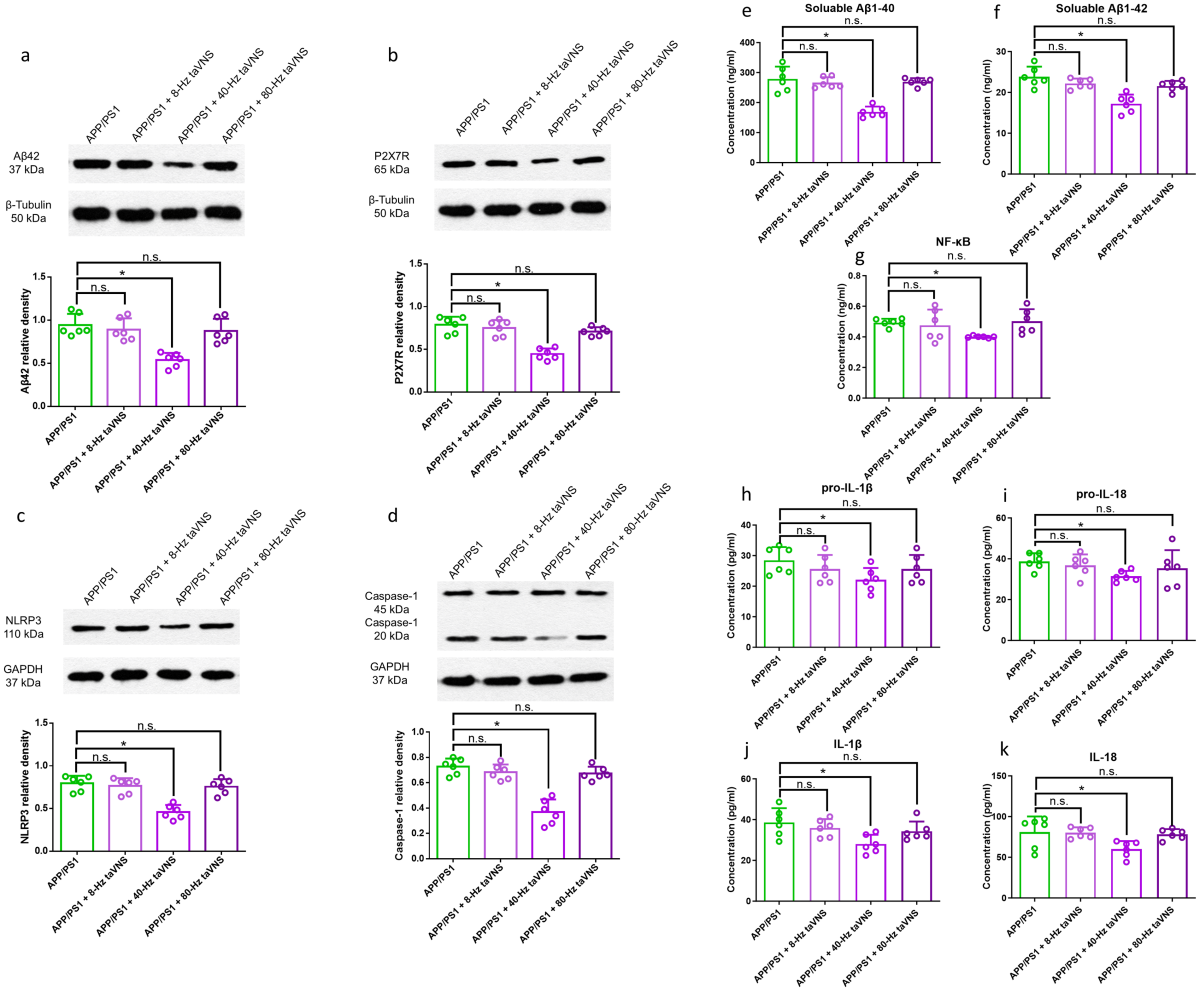
Hippocampal Aβ42, P2X7R, NLRP3, caspase-1 expression and Aβ1-40, Aβ1-42, NF-κB, pro-IL-1β, pro-IL-18, IL-1β, IL-18 levels in Part 2 of the study. WB results show hippocampal Aβ42 **(a)**, P2X7R **(b)**, NLRP3 **(c)**, and caspase-1 (20 kDa) **(d)** expression. *n* = 6 per group, APP/PS1 vs. APP/PS1 + 8-Hz taVNS and APP/PS1 vs. APP/PS1 + 80-Hz taVNS, n.s., not significant, *p* > 0.05; APP/PS1 vs. APP/PS1 + 40-Hz taVNS, *p < 0.05. ELISA results show soluble Aβ1-40 **(e)**, soluble Aβ1-42 **(f)**, NF-κB **(g)**, pro-IL-1β **(h)**, pro-IL-18 **(i)**, IL-1β **(j)**, and IL-18 **(k)** levels in the hippocampi of the mice. *n* = 6 per group, APP/PS1 vs. APP/PS1 + 8-Hz taVNS and APP/PS1 vs. APP/PS1 + 80-Hz taVNS, n.s., not significant, *p* > 0.05; APP/PS1 vs. APP/PS1 + 40-Hz taVNS, *p < 0.05.

Consistent with behavioral improvements, 40-Hz taVNS selectively decreased hippocampal Aβ42 levels in APP/PS1 mice, while 8-Hz and 80-Hz treatments showed no effect (Figure 5a). Similar trends were observed for soluble Aβ1-40 and Aβ1-42 (Figures 5e,f).

#### 40-Hz taVNS specifically inhibits P2X7R signaling in APP/PS1 mice aged 9 months

Mechanistically, 40-Hz taVNS uniquely suppressed the P2X7R signaling pathway, as evidenced by reduced expression of P2X7R, NLRP3, and Caspase-1 (20 kDa) (Figures 5b–d). Downstream inflammatory mediators, including NF-κB, pro-IL-1β/IL-1β, and pro-IL-18/IL-18, were also selectively downregulated by 40-Hz taVNS (Figures 5g–k). No such effects were found by 8-Hz and 80-Hz treatments (Figures 5b–d,g).

#### The neuroprotective benefits of both 8-Hz taVNS and 80-Hz taVNS are relatively weak

Nissl staining revealed that 40-Hz taVNS induced the most pronounced neuroprotective effects, with darker and more organized Nissl bodies in hippocampal cornu ammonis (CA)2–3, CA1, and dentate gyrus (DG) regions compared to 8-Hz or 80-Hz treatments (Supplementary Figure 2). This further supports the superiority of 40-Hz stimulation in preserving neuronal integrity.

### APP/PS1 mice at 9 months of age regain cognition by inhibiting P2X7R signaling with 40-Hz taVNS

#### Inhibiting P2X7R rescues cognition of APP/PS1 mice at 9 months of age while 40-Hz taVNS counteracts the cognition-worsening effects of P2X7R activating

**Figure 6:**
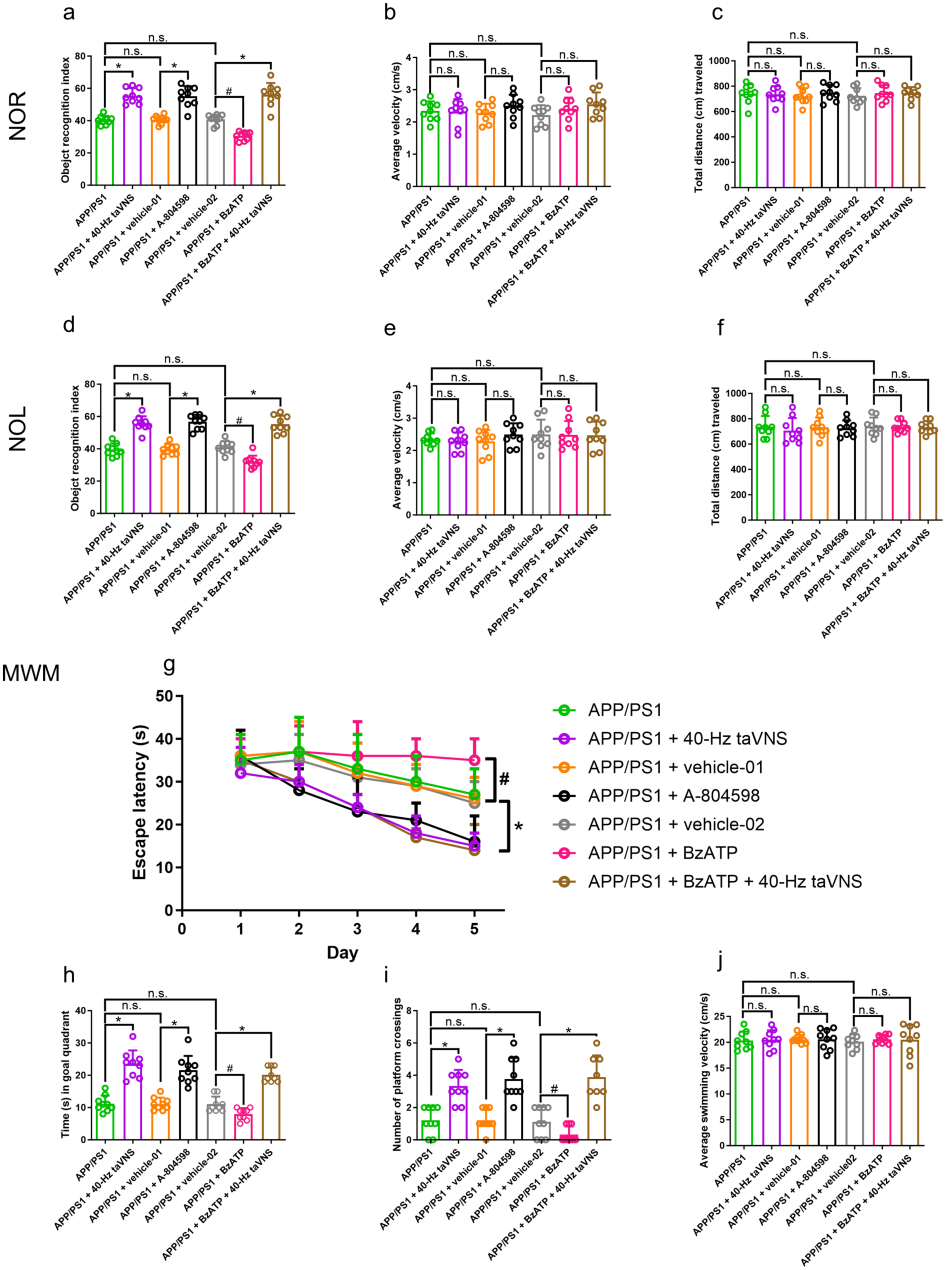
The results of the NOR, NOL, and MWM tests in part 3 of the study. **(a)** The recognition index of the NOR test (*n* = 9 per group, APP/PS1 vs. APP/PS1 + 40-Hz taVNS, APP/PS1 + vehicle-01 vs. APP/PS1 + A-804598, and APP/PS1 + vehicle-02 vs. APP/PS1 + BzATP + 40-Hz taVNS, *p < 0.05; APP/PS1 + vehicle-02 vs. APP/PS1 + BzATP, #*p* < 0.05; APP/PS1 vs. APP/PS1 + vehicle-01 and APP/PS1 vs. APP/PS1 + vehicle-02, n.s., not significant, *p* > 0.05). **(b)** The average velocity (cm/s), during the NOR test (*n* = 9 per group, APP/PS1 vs. APP/PS1 + vehicle-01, APP/PS1 vs. APP/PS1 + vehicle-02, APP/PS1 vs. APP/PS1 + 40-Hz taVNS, APP/PS1 + vehicle-01 vs. APP/PS1 + A-804598, APP/PS1 + vehicle-02 vs. APP/PS1 + BzATP, and APP/PS1 + vehicle-02 vs. APP/PS1 + BzATP + 40-Hz taVNS, n.s., not significant, *p* > 0.05). **(c)** The cumulative distance (cm) traveled throughout the NOR test (*n* = 9 per group, APP/PS1 vs. APP/PS1 + vehicle-01, APP/PS1 vs. APP/PS1 + vehicle-02, APP/PS1 vs. APP/PS1 + 40-Hz taVNS, APP/PS1 + vehicle-01 vs. APP/PS1 + A-804598, APP/PS1 + vehicle-02 vs. APP/PS1 + BzATP, and APP/PS1 + vehicle-02 vs. APP/PS1 + BzATP + 40-Hz taVNS, n.s., not significant, *p* > 0.05). **(d)** The recognition index of the NOL test (*n* = 9 per group, APP/PS1 vs. APP/PS1 + 40-Hz taVNS, APP/PS1 + vehicle-01 vs. APP/PS1 + A-804598, and APP/PS1 + vehicle-02 vs. APP/PS1 + BzATP + 40-Hz taVNS, *p < 0.05; APP/PS1 + vehicle-02 vs. APP/PS1 + BzATP, #*p* < 0.05; APP/PS1 vs. APP/PS1 + vehicle-01 and APP/PS1 vs. APP/PS1 + vehicle-02, n.s., not significant, *p* > 0.05). **(e)** The average velocity (cm/s), during the NOL test (*n* = 9 per group, APP/PS1 vs. APP/PS1 + vehicle-01, APP/PS1 vs. APP/PS1 + vehicle-02, APP/PS1 vs. APP/PS1 + 40-Hz taVNS, APP/PS1 + vehicle-01 vs. APP/PS1 + A-804598, APP/PS1 + vehicle-02 vs. APP/PS1 + BzATP, and APP/PS1 + vehicle-02 vs. APP/PS1 + BzATP + 40-Hz taVNS, n.s., not significant, *p* > 0.05). **(f)** The cumulative distance (cm) traveled throughout the NOL test (*n* = 9 per group, APP/PS1 vs. APP/PS1 + vehicle-01, APP/PS1 vs. APP/PS1 + vehicle-02, APP/PS1 vs. APP/PS1 + 40-Hz taVNS, APP/PS1 + vehicle-01 vs. APP/PS1 + A-804598, APP/PS1 + vehicle-02 vs. APP/PS1 + BzATP, and APP/PS1 + vehicle-02 vs. APP/PS1 + BzATP + 40-Hz taVNS, n.s., not significant, *p* > 0.05). **(g)** Escape latencies in the MWM (*n* = 9 per group, APP/PS1 vs. APP/PS1 + 40-Hz taVNS, APP/PS1 + vehicle-01 vs. APP/PS1 + A-804598, and APP/PS1 + vehicle-02 vs. APP/PS1 + BzATP + 40-Hz taVNS, *p < 0.05; APP/PS1 + vehicle-02 vs. APP/PS1 + BzATP, #*p* < 0.05). **(h)** Time spent swimming in the goal quadrant during the probe trial (*n* = 9 per group, APP/PS1 vs. APP/PS1 + 40-Hz taVNS, APP/PS1 + vehicle-01 vs. APP/PS1 + A-804598, and APP/PS1 + vehicle-02 vs. APP/PS1 + BzATP + 40-Hz taVNS, *p < 0.05; APP/PS1 + vehicle-02 vs. APP/PS1 + BzATP, #*p* < 0.05; APP/PS1 vs. APP/PS1 + vehicle-01 and APP/PS1 vs. APP/PS1 + vehicle-02, n.s., not significant, *p* > 0.05). **(i)** Number of platform crossings during the probe trial (*n* = 9 per group, APP/PS1 vs. APP/PS1 + 40-Hz taVNS, APP/PS1 + vehicle-01 vs. APP/PS1 + A-804598, and APP/PS1 + vehicle-02 vs. APP/PS1 + BzATP + 40-Hz taVNS, *p < 0.05; APP/PS1 + vehicle-02 vs. APP/PS1 + BzATP, #*p* < 0.05; APP/PS1 vs. APP/PS1 + vehicle-01 and APP/PS1 vs. APP/PS1 + vehicle-02, n.s., not significant, *p* > 0.05). **(j)** Average swimming velocity (cm/s) during MWM (*n* = 9 per group, APP/PS1 vs. APP/PS1 + vehicle-01, APP/PS1 vs. APP/PS1 + vehicle-02, APP/PS1 vs. APP/PS1 + 40-Hz taVNS, APP/PS1 + vehicle-01 vs. APP/PS1 + A-804598, APP/PS1 + vehicle-02 vs. APP/PS1 + BzATP, and APP/PS1 + vehicle-02 vs. APP/PS1 + BzATP + 40-Hz taVNS, n.s., not significant, *p* > 0.05).

The NOR index, NOL index, escape latency, time in the goal quadrant, and number of platform crossings were not remarkably different between the APP/PS1, APP/PS1 + vehicle-01, and APP/PS1 + vehicle-02 groups (Figures 6a,d,g,h,i).

Pharmacological inhibition of P2X7R with A-804598 mimicked the cognitive benefits of 40-Hz taVNS (Figures 6a,d,g,h,i). In contrast, P2X7R activation via BzATP exacerbated cognitive deficits, which were fully rescued by co-administration of 40-Hz taVNS (Figures 6a,d,g,h,i). Locomotor activity was unchanged across all groups (Figures 6b,c,e,f,j).

#### Inhibiting P2X7R reduces the accumulation of amyloid in APP/PS1 mice at 9 months of age while 40-Hz taVNS counteracts the amyloid-accumulating effects of P2X7R activating

**Figure 7:**
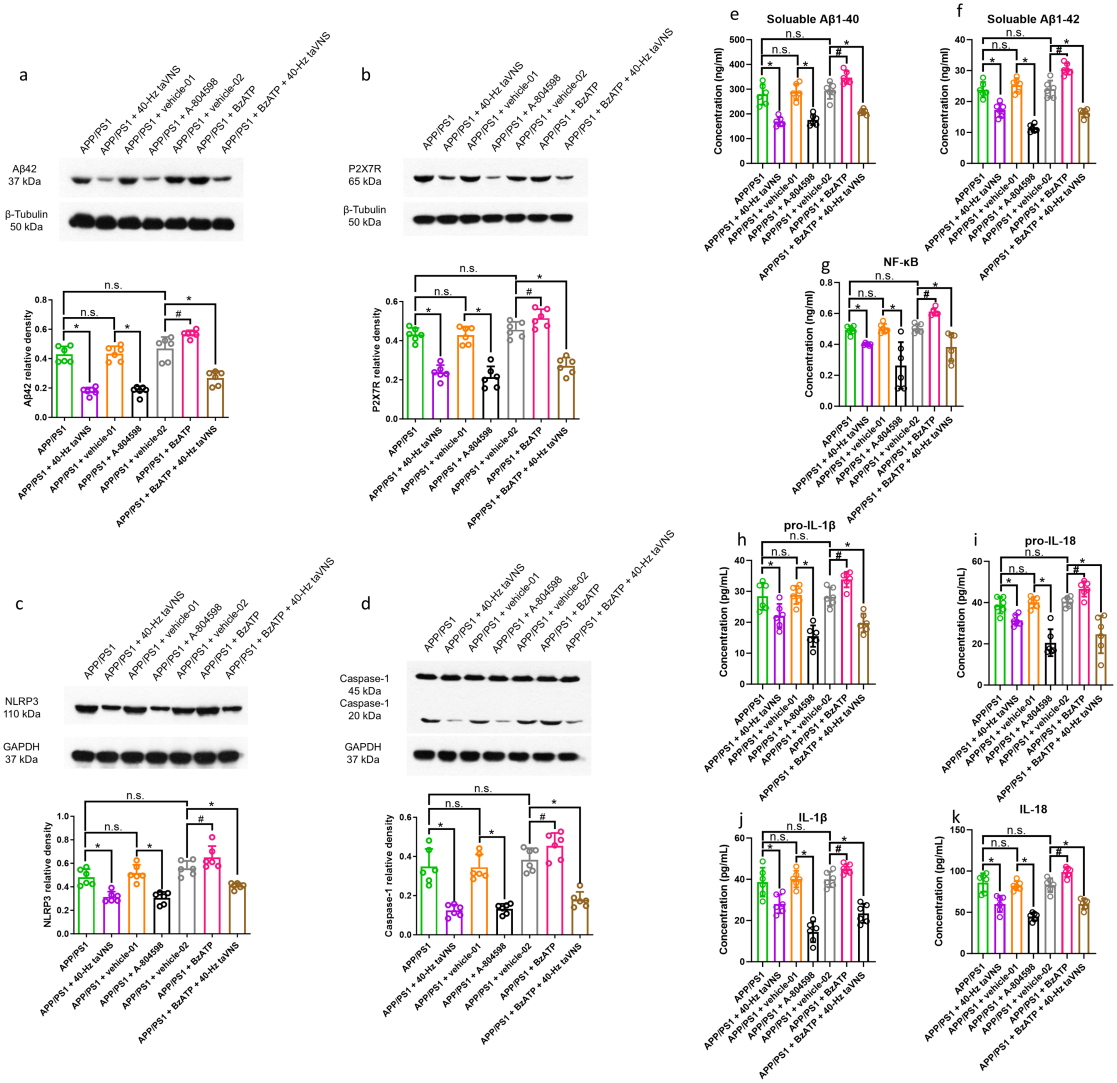
Hippocampal Aβ42, P2X7R, NLRP3, Caspase-1 expression, and Aβ1-40, Aβ1-42, NF-κB, pro-IL-1β, pro-IL-18, IL-1β, IL-18 levels in part 3 of the study. WB results show hippocampal Aβ42 **(a)**, P2X7R **(b)**, NLRP3 **(c)**, and Caspase-1 (20 kDa) **(d)** expression (*n* = 6 per group, APP/PS1 vs. APP/PS1 + 40-Hz taVNS, APP/PS1 + vehicle-01 vs. APP/PS1 + A-804598, and APP/PS1 + vehicle-02 vs. APP/PS1 + BzATP + 40-Hz taVNS, *p < 0.05; APP/PS1 + vehicle-02 vs. APP/PS1 + BzATP, #*p* < 0.05; APP/PS1 vs. APP/PS1 + vehicle-01 and APP/PS1 vs. APP/PS1 + vehicle-02, n.s., not significant, *p* > 0.05. ELISA results show soluble Aβ1-40 **(e)**, soluble Aβ1-42 **(f)**, NF-κB **(g)**, pro-IL-1β **(h)**, pro-IL-18 **(i)**, IL-1β **(j)**, and IL-18 **(k)** levels in the hippocampi of the mice. *n* = 6 per group, APP/PS1 vs. APP/PS1 + 40-Hz taVNS, APP/PS1 + vehicle-01 vs. APP/PS1 + A-804598, and APP/PS1 + vehicle-02 vs. APP/PS1 + BzATP + 40-Hz taVNS, *p < 0.05; APP/PS1 + vehicle-02 vs. APP/PS1 + BzATP, #*p* < 0.05; APP/PS1 vs. APP/PS1 + vehicle-01, and APP/PS1 vs. APP/PS1 + vehicle-02, n.s., not significant, *p* > 0.05).

The APP/PS1, APP/PS1 + vehicle-01, and APP/PS1 + vehicle-02 groups exhibited no significant difference in hippocampal Aβ42 expression as well as soluble Aβ1-40 and Aβ1-42 levels (Figures 7a,e,f). BzATP treatment significantly increased hippocampal Aβ42 levels, whereas both 40-Hz taVNS and A-804598 reduced Aβ42 accumulation, with 40-Hz taVNS being able to reverse the effects of BzATP (Figure 7a). Similar trends were observed for hippocampal soluble Aβ1-40 and Aβ1-42 (Figures 7e,f).

#### A-804598 inhibits P2X7R signaling in APP/PS1 mice at 9 months of age while 40-Hz taVNS counteracts P2X7R-signaling-activating effects by BzATP

Mice in the APP/PS1, APP/PS1 + vehicle-01, and APP/PS1 + vehicle-02 groups exhibited no significant alterations in hippocampal concentrations of P2X7R, NLRP3, and Caspase-1 (20 kDa) (Figures 7b–d) and in hippocampal levels of NF-κB, pro-IL-1β/IL-1β, and pro-IL-18/IL-18 (Figures 7g–k).

P2X7R activation by BzATP upregulated hippocampal expression of P2X7R, NLRP3, and Caspase-1 (20 kDa), key mediators of neuroinflammation (Figures 7b–d). Conversely, 40-Hz taVNS and A-804598 suppressed the P2X7R signaling pathway, with 40-Hz taVNS achieving a near-complete reversal of BzATP-induced effects (Figures 7b–d). Downstream inflammatory markers (NF-κB, pro-IL-1β/IL-1β, and pro-IL-18/IL-18) followed the same pattern (Figures 7g–k).

#### The neuroprotective benefits of A-804598 are weaker than 40-Hz taVNS

Nissl staining revealed that 40-Hz taVNS provided superior neuroprotection compared to A-804598, as evidenced by denser Nissl bodies in hippocampal neurons (Supplementary Figure 3). These findings suggest that the neuroprotective properties of A-804598 are comparatively less potent when compared to the effects of taVNS at 40 Hz.

## Discussion

Our team pioneered 40-Hz taVNS in AD models (Yu et al., 2023), inspired by Tsai’s gamma stimulation research (Iaccarino et al., 2016). Subsequent studies on multi-sensory gamma entrainment (Adaikkan et al., 2019; Martorell et al., 2019; Murdock et al., 2024) highlighted its potential, leveraging the vagal nerve as a conduit for interoceptive signals (Craig, 2002). This approach combines gamma entrainment (GENUS) with vagal modulation via ABVN, offering a non-invasive brain stimulation treatment for AD. While our prior work showed efficacy in early-stage AD mice (Yu et al., 2023), the current study extends these findings by demonstrating cognitive rescue even in advanced AD, establishing its therapeutic potential across disease stages.

APP/PS1 mice, a common transgenic model of AD, exhibit stable amyloid deposition by 6 months of age (Jankowsky et al., 2004) and develop age-related cognitive deficits, with spatial memory impairment preceding recognition memory decline (Woo et al., 2010; Webster et al., 2013). Our previous study showed that 6-month-old APP/PS1 mice have selective spatial learning and memory deficits without recognition impairment (Yu et al., 2023), whereas the present study reveals that 9-month-old mice display significant impairments in both spatial and recognition memory, indicating profound overall cognitive decline.

This research elucidated the impact of different interventions on cognition in 9-month-old APP/PS1 mice. Part 1 revealed that 40 Hz taVNS rescued cognition in APP/PS1 mice but not in age-matched WT mice. Part 2 demonstrated that these cognitive benefits were frequency-dependent, specifically at 40 Hz. Part 3 illustrated that the rescuing effect of 40 Hz taVNS in APP/PS1 mice was mediated by regulation of the P2X7R signaling pathway.

The extracellular deposition of Aβ plaques is a hallmark of AD. Two main Aβ isoforms, Aβ1-40 and Aβ1-42, contribute differently to disease progression. BRI-Aβ42 mice, despite producing tenfold less Aβ1-42 than BRI-Aβ40 mice produce Aβ1-40, develop amyloid deposits by 3 months of age, whereas BRI-Aβ40 mice show no amyloid pathology (McGowan et al., 2005). Research indicates that Aβ1-42 promotes amyloid accumulation, while Aβ1-40 inhibits it (Kim et al., 2007). One proposed mechanism is that Aβ1-40 competes for binding sites on Aβ1-42 aggregates, thereby limiting further aggregation (Murray et al., 2009a; Murray et al., 2009b).

This work demonstrated that 40-Hz taVNS significantly reduced hippocampal Aβ1-42 and Aβ1-40 levels in 9-month-old APP/PS1 mice, an effect dependent on frequency (40 Hz being effective). Additionally, both P2X7R inhibition and 40-Hz taVNS suppressed hippocampal Aβ, with 40-Hz taVNS counteracting P2X7R activation.

Among Aβ isoforms, Aβ40 is predominant, followed by Aβ42 (Luo et al., 2020). However, Aβ42 is hydrophobic and aggregates faster than Aβ40 (Barage and Sonawane, 2015). Co-expression of APP and PS1 selectively elevates Aβ42 without affecting Aβ40 (Jankowsky et al., 2004). Aβ42 exhibits significant neurotoxicity, leading to neuronal damage and loss (Perl, 2010).

Part 1 of this work revealed that 40-Hz taVNS significantly decreased hippocampal Aβ42 in APP/PS1 mice but not in WT mice. Part 2 demonstrated the frequency-specific (40 Hz) effect of taVNS on Aβ42. Part 3 illustrated that inhibiting P2X7R and 40-Hz taVNS suppressed Aβ42, while 40-Hz taVNS counteracted the effect of P2X7R activation on Aβ42.

Overall, the findings from the analysis of Aβ1-40, Aβ1-42, and Aβ42 measurement demonstrate that the use of 40-Hz taVNS exclusively decreases the quantity of Aβ. These findings demonstrate that the application of taVNS at 40 Hz can reduce the accumulation of amyloid in APP/PS1 mice at 9 months of age, with P2X7R signaling participating in the process.

Aβ induces neuroinflammation by altering P2X7R activity (Sanz et al., 2009). P2X7R activation drives neuroinflammation via the NLRP3-Caspase-1 pathway, and NLRP3 inflammasome activation is essential for initiating neurodegeneration (Thawkar and Kaur, 2019). Thus, in both our previous (Yu et al., 2023) and current studies, we propose that 40 Hz taVNS may directly attenuate neuroinflammation by suppressing P2X7R signaling, thereby reversing AD-associated pathology.

In this study, we identified P2X7R, NLRP3, and Caspase-1 in the hippocampi. Part 1 data suggested that activation of the hippocampal P2X7R pathway exacerbates cognitive decline in 9-month-old APP/PS1 mice, and that 40-Hz taVNS effectively reversed these changes. Part 2 showed that this corrective effect was specific to 40-Hz stimulation. Part 3 directly demonstrated that inhibiting the hippocampal P2X7R pathway is crucial for the cognition-rescuing effects of 40-Hz taVNS.

Pro-IL-1β and pro-IL-18 are dormant precursors of IL-1β and IL-18 found in the cytoplasm. They are produced by the NF-κB signaling pathway and play a crucial role in the P2X7R signaling pathway (Hanslik and Ulland, 2020; Chai et al., 2022).

The Part 1 results revealed that taVNS at a frequency of 40 Hz inhibits the P2X7R signaling pathway in the hippocampi of the APP/PS1 mice. The Part 2 results demonstrated the frequency specificity of taVNS (40 Hz) in APP/PS1 mice at 9 months of age. The Part 3 results offered conclusive evidence indicating that the observed effectiveness of taVNS at a frequency of 40 Hz in APP/PS1 mice aged 9 months can be ascribed to the alteration of the P2X7R signaling pathway.

In the field of cellular neuroscience, Nissl bodies are distinct granular structures found in neurons. Their name is derived from Franz Nissl who is credited with inventing the staining technique (Gomes, 2019). Nissl bodies exhibit alterations in response to different physiological states and pathological circumstances, such as axonotmesis, in which they may undergo dissolution and significant reduction (chromatolysis). If the neuron successfully repairs the injury, the Nissl bodies eventually reemerge and resume their characteristic distribution inside the cell (Kreutzberg, 1984; Luo et al., 2020). Therefore, the appearance of Nissl bodies can be a sign of neuroprotective effects by interventions.

The images from Part 1 revealed that taVNS at 40 Hz is involved in facilitating neuroprotective effects, which ultimately leads to the restoration of cognition in APP/PS1 mice at 9 months of age. The images from Part 2 demonstrated that taVNS at 40 Hz processes more potent neuroprotective effects than 8-Hz taVNS and 80-Hz taVNS. The images from Part 3 indicated that the neuroprotective effects of 40-Hz taVNS are stronger than other interventions.

Here, we establish a series of comparisons between the present study and our prior work (Yu et al., 2023). First, the current investigation revealed that APP/PS1 mice at 9 months of age exhibited deficiencies in recognition, spatial learning, and memory abilities, indicating a complete impairment in cognition. Concurrently, we may conclude from the current study that 40-Hz taVNS effectively rescues cognition of APP/PS1 mice aged 9 months, a result that was unattainable in our earlier research (Yu et al., 2023). Second, we used a solitary stimulating frequency (40 Hz) in our previous work (Yu et al., 2023), and two additional (8 Hz and 80 Hz) were incorporated in this study, inspired by Tsai and colleagues (Martorell et al., 2019). The results confirmed that 40 Hz stimulation works best. Third, the use of P2X7R antagonist and agonist in this work revealed that taVNS at 40 Hz rescues the cognition of APP/PS1 mice aged 9 months via inhibiting hippocampal P2X7R signaling pathway (causation), which was lacking verification previously (Yu et al., 2023).

The current investigation addressed some inquiries that were raised in the preceding study (Yu et al., 2023); however, it still encountered certain constraints. First, sex was not considered as a variable in the experimental design; future studies should consider the use female mice. Second, taVNS’s efficiency may be affected by anesthetics, but rodents’ intolerance prevents better possibilities; future studies should aim to validate these findings in awake animal models. Third, as 20-Hz or 100-Hz may work in MCI patients (Wang et al., 2022), more frequencies should be examined in future work, including clinically relevant bands like 20–30 Hz. Fourth, if technically feasible and adequately funded, APP/PS1xP2X7Rko mice may be a better choice than APP/PS1 mice administrated with P2X7R antagonists (Martin et al., 2019), APP/PS1xP2X7Rki mice (P2X7R overexpression) should also be considered as the alternative of APP/PS1 + BzATP and APP/PS1 + BzATP + 40-Hz taVNS groups; moreover, neuron/microglia-specific knockout techniques across with APP/PS1 mice should be considered in future works. Fifth, lacking of microglial-specific markers (Iba1, CD68) and pyroptosis markers (Gasdermin D) limits our ability to pinpoint the cellular source of the inflammasome activation. Sixth, future studies should include motor and anxiety controls to ensure that the cognitive results are not confounded by these factors. Seventh, long-term data (more than 16-day treatment and longer-term efficacy tests) should be considered in future works. Eighth, this study may be the first to use a P2X7R antagonist in APP/PS1 mice and find promising outcomes (Territo and Zarrinmayeh, 2021; Huang et al., 2023), yet lacking further verification. More AD models and P2X7R antagonists should be used to confirm this crucial finding.

## Conclusion

**Figure 8:**
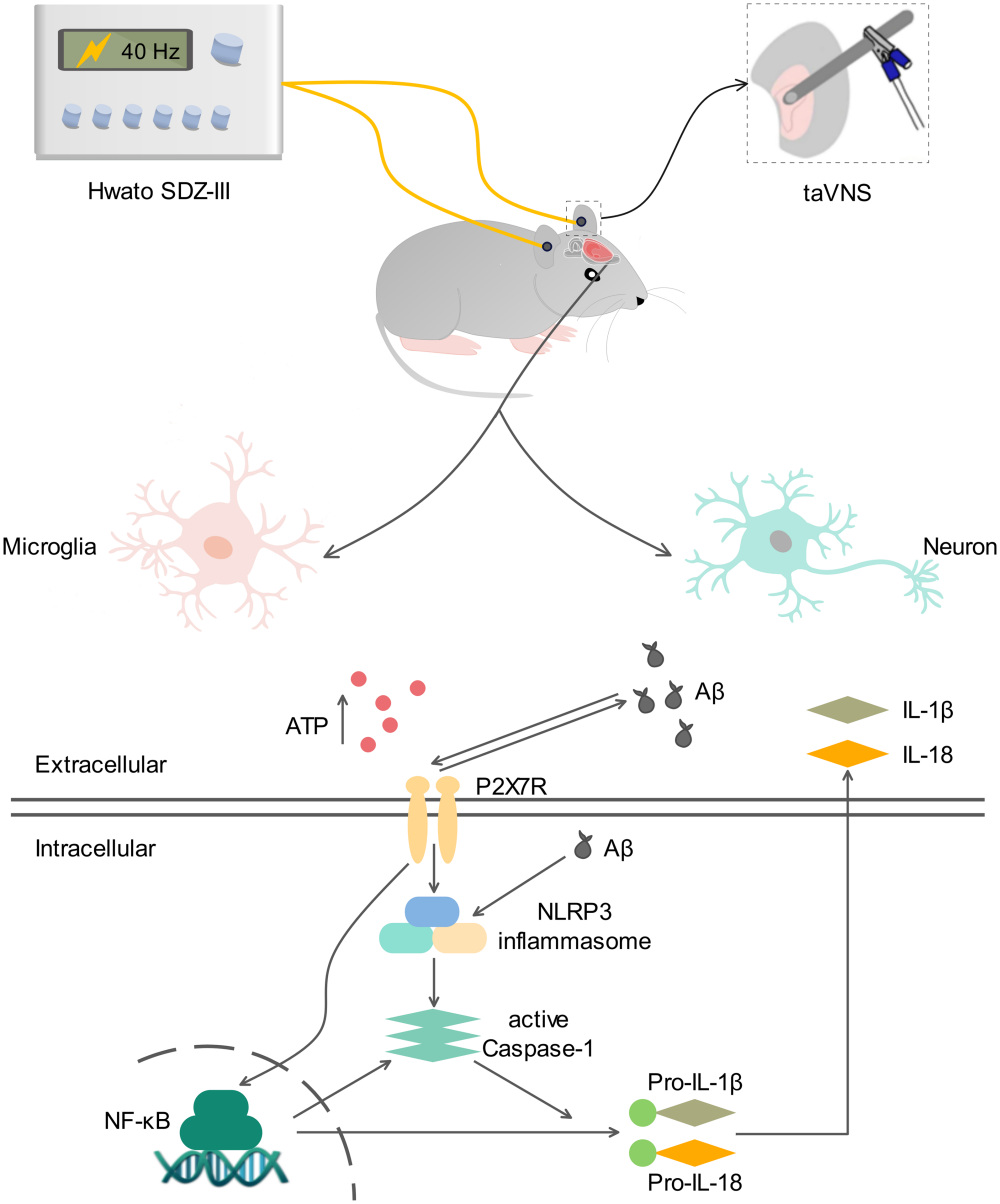
Illustration of the mechanisms interpreted by this study. Stimulation of the auricular branch of the vagal nerve (ABVN) at a frequency of 40 Hz (40-Hz taVNS) inhibits the signaling pathway of the P2X7R, resulting in the suppressive interactions between P2X7R and Aβ, P2X7R and NLRP3, and P2X7R and NF-κB. This leads to a downregulation of the inflammatory cascade, including the mediators of P2X7R, thereby reducing inflammatory responses. As a result, the pathological features associated with AD are reversed, and neuronal restoration is promoted. Ultimately, it rescues the cognition of 9-month-old APP/PS1 mice.

In summary, the current investigation deepened our insight into 40-Hz taVNS in ameliorating AD compared to previous work (Figure 8). We can draw three conclusions from the study. (1) 40-Hz taVNS rescues cognition of 9-month-old APP/PS1 mice. (2) The cognition-rescuing effects of taVNS in APP/PS1 mice at 9 months of age are frequency-specific, which is 40 Hz in this work. (3) The hippocampal P2X7R signaling is a critical mediator of the observed effects. The results of this work indicate that taVNS with a frequency of 40 Hz may be a favorable therapeutic alternative for AD and that P2X7R may be a specific target, necessitating additional investigation in both alternative AD models and diagnosed AD patients.

## Data Availability

The datasets presented in this study can be found in online repositories. The names of the repository/repositories and accession number(s) can be found in the article/Supplementary material.

## Footnotes

1
www.geneandpeace.com

## Supplementary material

SUPPLEMENTARY FIGURE 1 Histological assessments in Part 1 of the study. Representative illustrations depicting Nissl staining in the hippocampi of the WT, WT + 40-Hz taVNS, APP/PS1, and APP/PS1 + 40-Hz taVNS groups. Bar, 100 μm.

SUPPLEMENTARY FIGURE 2 Histological assessments in Part 2 of the study. Representative illustrations depicting Nissl staining in the hippocampi of the APP/PS1, APP/PS1 + 8-Hz taVNS, APP/PS1 + 40-Hz taVNS, and APP/PS1 + 80-Hz taVNS groups. Bar, 100 μm.

SUPPLEMENTARY FIGURE 3 Histological assessments in Part 3 of the study. Representative illustrations depicting Nissl staining in the hippocampi of the APP/PS1, APP/PS1 + 40-Hz taVNS, APP/PS1 + vehicle-01, APP/PS1 + A-804598, APP/PS1 + vehicle-02, APP/PS1 + BzATP, and APP/PS1 + BzATP+ 40-Hz taVNS groups. Bar, 100 μm.
