# Supplementary figures and images for: 40-Hz transauricular vagal nerve stimulation rescues cognition of 9-month-old APP/PS1 mice via inhibiting hippocampal P2X7 receptor signaling
Source: Front Aging Neurosci. 2026 Mar 13;18:1766813. doi: 10.3389/fnagi.2026.1766813 (PMC13021832; doi:10.3389/fnagi.2026.1766813)

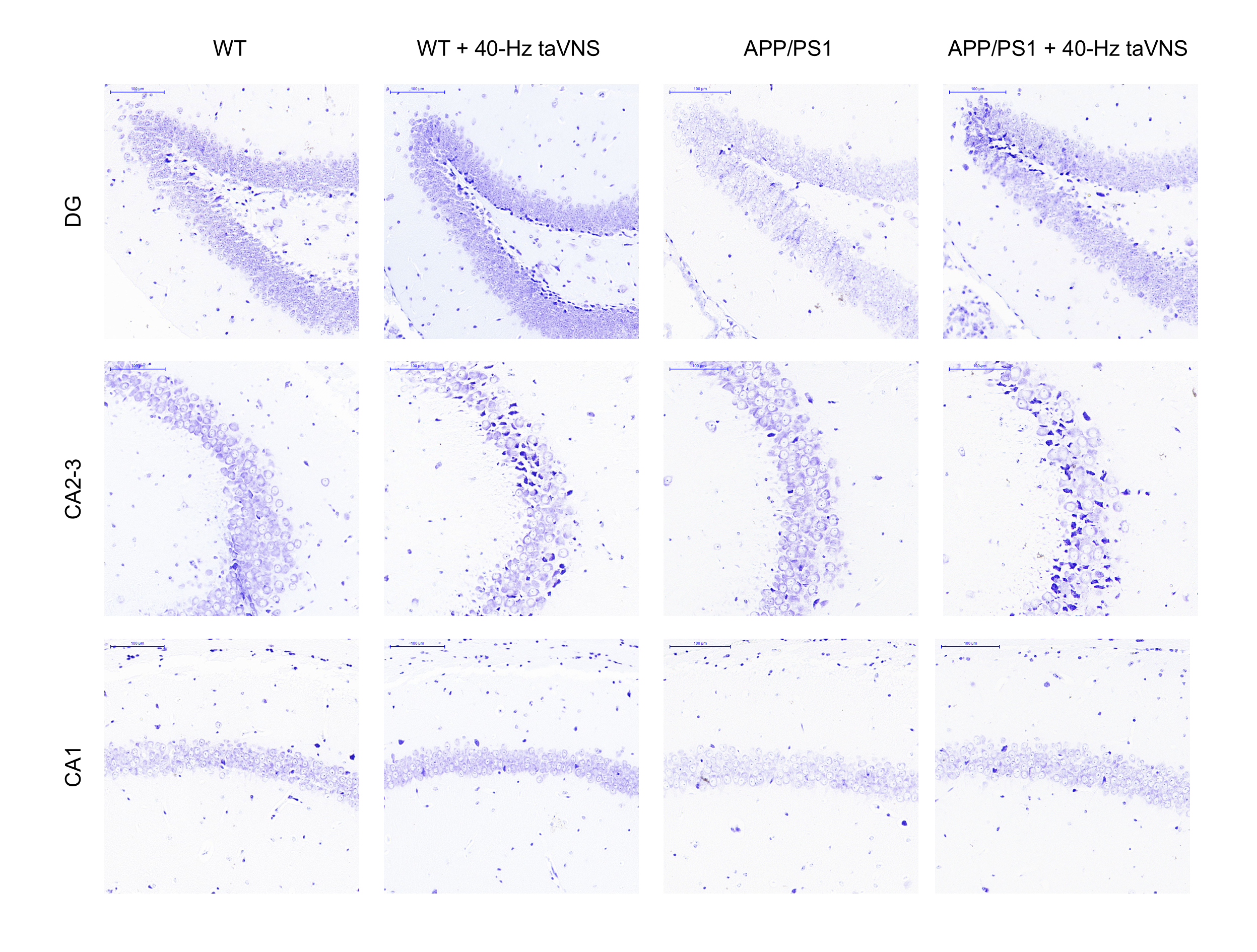

Supplement: SUPPLEMENTARY FIGURE 1 — Histological assessments in Part 1 of the study. Representative illustrations depicting Nissl staining in the hippocampi of the WT, WT + 40-Hz taVNS, APP/PS1, and APP/PS1 + 40-Hz taVNS groups. Bar, 100 μm. [file Image_1.tif]

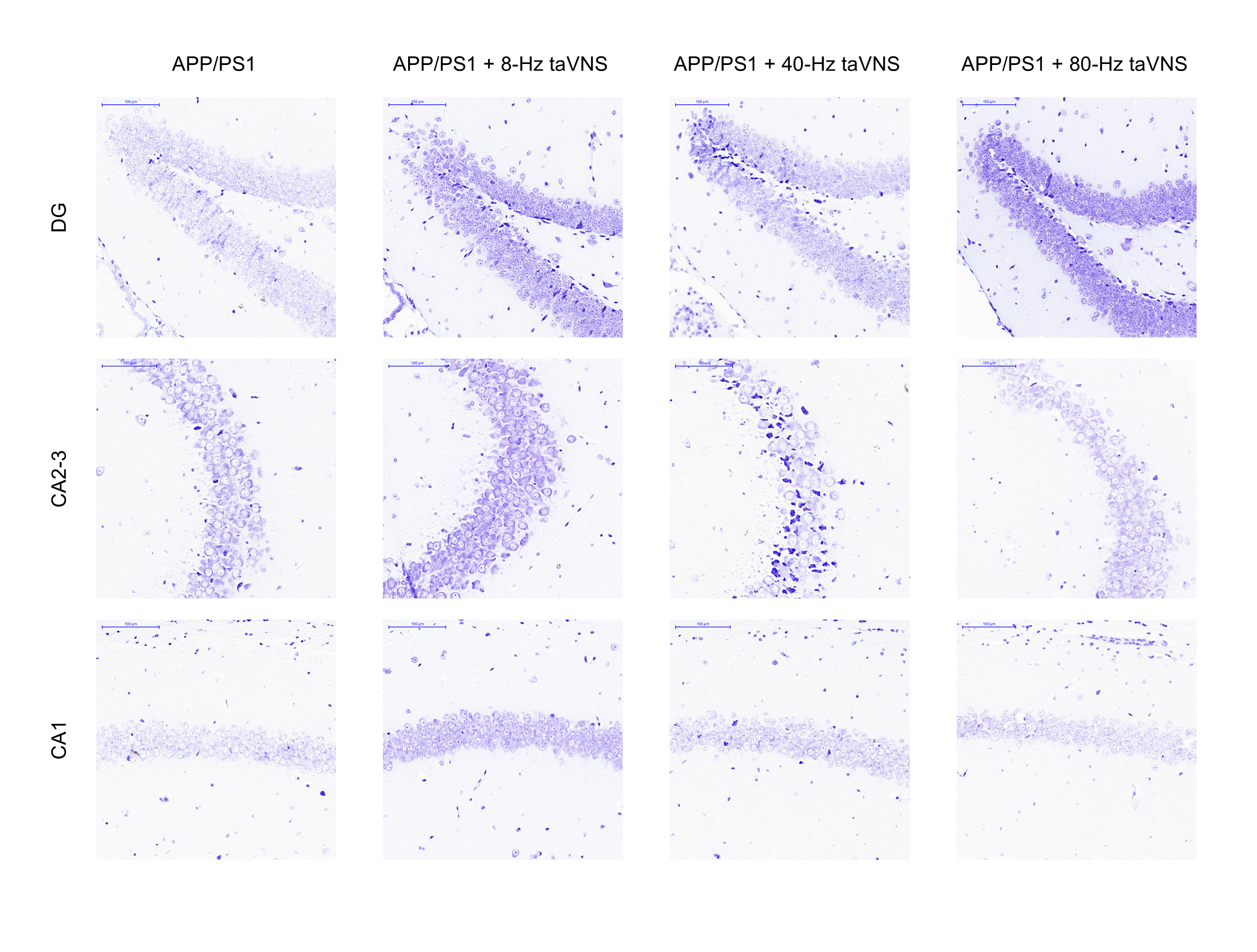

Supplement: SUPPLEMENTARY FIGURE 2 — Histological assessments in Part 2 of the study. Representative illustrations depicting Nissl staining in the hippocampi of the APP/PS1, APP/PS1 + 8-Hz taVNS, APP/PS1 + 40-Hz taVNS, and APP/PS1 + 80-Hz taVNS groups. Bar, 100 μm. [file Image_2.tif]

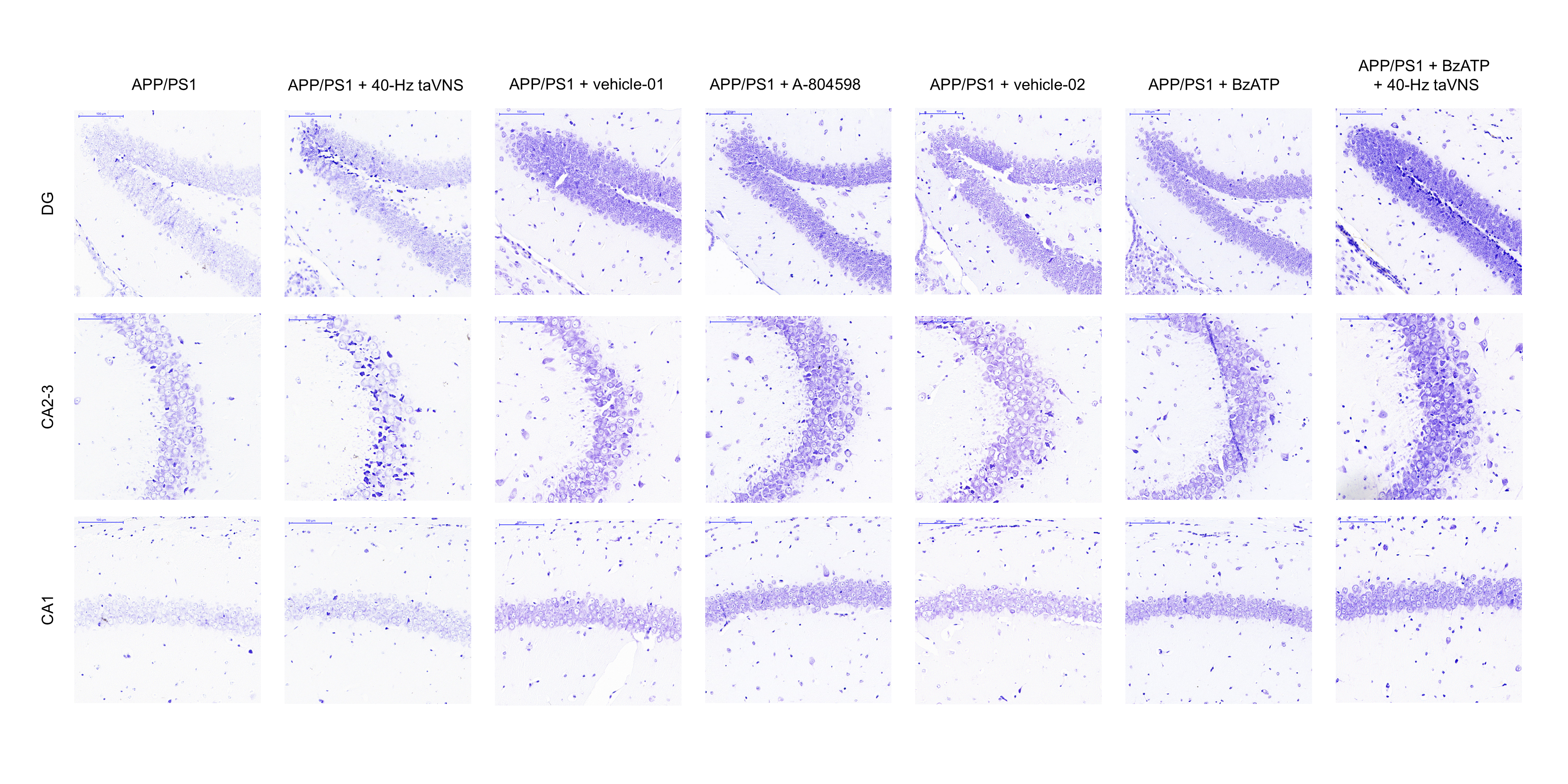

Supplement: SUPPLEMENTARY FIGURE 3 — Histological assessments in Part 3 of the study. Representative illustrations depicting Nissl staining in the hippocampi of the APP/PS1, APP/PS1 + 40-Hz taVNS, APP/PS1 + vehicle-01, APP/PS1 + A-804598, APP/PS1 + vehicle-02, APP/PS1 + BzATP, and APP/PS1 + BzATP+ 40-Hz taVNS groups. Bar, 100 μm. [file Image_3.tif]
